# Transcriptome analysis reveals potential mechanisms for different grain size between natural and resynthesized allohexaploid wheats with near-identical AABB genomes

**DOI:** 10.1186/s12870-018-1248-y

**Published:** 2018-02-05

**Authors:** Lei Yan, Zhenshan Liu, Huanwen Xu, Xiaoping Zhang, Aiju Zhao, Fei Liang, Mingming Xin, Huiru Peng, Yingyin Yao, Qixin Sun, Zhongfu Ni

**Affiliations:** 1 State Key Laboratory for Agrobiotechnology, Key Laboratory of Crop Heterosis and Utilization (MOE), Beijing Key Laboratory of Crop Genetic Improvement, China Agricultural University, Beijing, 100193 China; 2 State Key Laboratory of Crop Stress Biology for Arid Areas, College of Agronomy, Northwest A&F University, Yangling, Shaanxi 712100 China; 3 Hebei Crop Genetic Breeding Laboratory Institute of Cereal and Oil Crops, Hebei Academy of Agriculture and Forestry Sciences, Shijiazhuang, 050035 China

**Keywords:** Allohexaploid wheat, D genome, Grain size and weight, Gene expression

## Abstract

**Background:**

Common wheat is a typical allohexaploid species (AABBDD) derived from the interspecific crossing between allotetraploid wheat (AABB) and *Aegilops tauschii* (DD). Wide variation in grain size and shape observed among *Aegilops tauschii* can be retained in synthetic allohexaploid wheats, but the underlying mechanism remains enigmatic. Here, the natural and resynthesized allohexaploid wheats with near-identical AB genomes and different D genomes (TAA10 and XX329) were employed for analysis.

**Results:**

Significant differences in grain size and weight between TAA10 and XX329 were observed at the early stages of development, which could be mainly attributed to the higher growth rates of the pericarp and endosperm cells in XX329 compared to TAA10. Furthermore, comparative transcriptome analysis identified that 8891 of 69,711 unigenes (12.75%) were differentially expressed between grains at 6 days after pollination (DAP) of TAA10 and XX329, including 5314 up-regulated and 3577 down-regulated genes in XX329 compared to TAA10. The MapMan functional annotation and enrichment analysis revealed that the differentially expressed genes were significantly enriched in categories of cell wall, carbohydrate and hormone metabolism. Notably, consistent with the up-regulation of sucrose synthase genes in resynthesized relative to natural allohexaploid wheat, the resynthesized allohexaploid wheat accumulated much higher contents of glucose and fructose in 6-DAP grains than those of the natural allohexaploid wheat.

**Conclusions:**

These data indicated that the genetic variation of the D genome induced drastic alterations of gene expression in grains of the natural and resynthesized allohexaploid wheats, which may contribute to the observed differences in grain size and weight.

**Electronic supplementary material:**

The online version of this article (10.1186/s12870-018-1248-y) contains supplementary material, which is available to authorized users.

## Background

Wheat is the leading food crop produced, consumed, and traded worldwide today. To meet the demand for burgeoning human population, we need wheat cultivars with higher yield potential [1]. The grain yield of wheat is mainly determined by the number of grains per m^2^ and grain weight. Modern breeding has greatly improved wheat yield by increasing grains per m^2^ due to the utilization of dwarfing genes (*Rht*) in 1960s and 1970s [2]. Analysis of 1800 cultivars exhibited that thousand grain weight (TGW) increased from a mean 31.5 g in the 1940s to 44.64 g in the 2000s, with an average 2.19 g increase in each decade, indicating that TGW is an important target for the improvement of wheat yield potential [3]. In the past two decades, the successful application of quantitative-genetic methodology has facilitated the identification of numerous QTL for TGW on all 21 wheat chromosomes [4–8]. Recently, several genes associated with wheat grain size and weight were isolated by using a homology-based approach, such as *TaCwi*, *TaGW2*, *TaTGW6*, *TaGS1a*, *TaGASR7-A1* and *TaCYP78A3* [9–15], which enhanced our knowledge about grain size and weight determination in wheat.

Grain development is an important and complex process in the wheat life cycle and directly affects the grain weight, which can be divided into five main phases: fertilization, “coenocytic” endosperm, cellularization and early grain filling, maximum grain filling, and desiccation [16]. At the level of gene expression, growing studies exhibited that the large changes in transcript/protein abundance were associated with distinct phases of wheat grain development. For example, novel distinct spatial gene expression patterns during wheat caryopsis development have been revealed by using a novel high-throughput mRNA in situ hybridization [17]. Distinct co-expression clusters reflecting the spatiotemporal progression during wheat endosperm development were identified by the cell type-specific expression analysis [18]. Although these studies provided a number of candidate genes important for grain growth and development, the molecular mechanisms involved in the determination of grain weight and size are still poorly understood.

Common wheat is a natural allohexaploid with A, B and D genomes contributed by *Triticum urartu*, a close relative of *Aegilops speltoides* and *Aegilops tauschii* (*syn. Ae. squarrosa, Tritcum tauschii*), respectively [19]. However, only a few *Aegilops tauschii*’s intraspecific lineages contributed to the evolution of common wheat, which resulted in limited D-genome variation represented in hexaploid bread wheat [20]. Thus, to increase the D-genome diversity in bread wheat, *Aegilops tauschii* has been used to introgress various traits of economic importance into bread wheat [21–24]. Specifically, wide variation in grain size and shape observed among *Ae. tauschii* accessions is retained in synthetic allohexaploid wheat lines [25–27]. Moreover, several studies have been carried out to identify beneficial QTL for grain weight from the diploid D donor of common wheat [28, 29]. For instance, a major QTL *QGw.caas-3D* controlling higher grain weight was identified on chromosome 3D of synthetic hexaploid wheat Am3 [29]. In 1964, an allotetraploid wheat (ETW) containing the AABB component was extracted from the allohexaploid bread wheat (TAA10). Afterwards, a resynthesized allohexaploid wheat (XX329) was produced by crossing ETW and the *Ae. tauschii subsp. strangulate* (TQ18). Interestingly, the grain size and weight of the resynthesized allohexaploid wheat (XX329) are much higher than that of TAA10 [30]. Since the AABB genomes of XX329 should be very similar to that of the donor TAA10, the observed variation in grain size and weight between these two genotypes may be mainly attributed to the differences of D genome, but the underlying molecular basis is still poorly understood.

The wheat caryopsis is a complex tissue in which maternal and endosperm tissues follow distinct but coordinated developmental programs [17]. During the initial phase of grain growth, maternal tissues (the pericarp of the grains) undergo a remarkable expansion as they are the main component of grain at this time. Thereby, grain size and weight determination are driven by pericarp growth [31]. Here, to dissect the underlying molecular basis for the variation of grain size and weight between natural and resynthesized allohexaploid wheats (TAA10 and XX329), the dynamic grain size and weight of these two genotypes were investigated, and grains at 6 DAP were selected for comparative transcriptome analysis. The results revealed that the variation in grain size could be attributed to the difference in pericarp expansion between TAA10 and XX329. Notably, the enrichment of differentially expressed genes involved in carbohydrate and cell wall metabolism may play important roles in the observed differences between TAA10 and XX329 in terms of grain size and weight.

## Methods

### Plant materials

The natural allohexaploid wheat TAA10 and the resynthesized allohexaploid wheat XX329 were grown in three biological replicates (5 rows/replicate) in Shangzhuang, Beijing (E116°, N40°) in the autumn of 2015. Grains were well distributed in rows that were 1.5 m long and 0.3 m apart with a sowing rate at 20 grains per row.

### Grain weight and volume measurements

Developing grains of TAA10 and XX329 for grain weight and volume measurements were collected at 2, 4, 6, 8, 10, 15, 20, 25, 30 and 35 days after pollination (DAP) with three biological replicates. In each replicate for each developmental stage, five spikes were sampled and the grains in eight central spikelets of each spike were used to measure grain fresh weight, dry weight and volume. The dry weight of grains was recorded after oven drying at 105 °C for 15 min and 65 °C for 48 h. The grain volume was determined using the “water displacement” principle, which was measured as the volume change of 95% alcohol after placing the grains in the measuring tube.

### Cytological observations and measurements

For cytological observations, the grains of TAA10 and XX329 at 2, 4, 6, 8, 10 and 15 DAP were collected with three biological replicates. Each replicate contained the grains in eight central spikelets of one spike. Grains from different samples were fixed in 50% (*v*/v) ethanol, 5% (v/v) acetic acid, and 3.7% (*w*/*v*) formaldehyde over 12 h at 4 °C, followed by dehydration and embedding in paraffin. Cross sections (3 μm) from the middle part of grains were cut using a Leica Ultracut rotary microtome and stained with Periodic acid Schiff (PAS). Photographs were taken with Pannoramic MIDI (3DHISTECH, Ltd., Hungary).

Cross sections of three grains for each developmental stage (2, 4, 6, 8, 10 and 15 DAP) were used to measure cell area. On each cross section, the cell area of the pericarp was measured by selecting three regions within five-row cells from the epicarp inwards, and the cell area of the endosperm was measured using three randomly selected regions of the endosperm. In each of the measured regions, between 20 and 40 cells were measured with CaseViewer 2.0 (3DHISTECH, Ltd., Hungary).

### Sugar content analysis

For sugar content analysis, the grains at 6 DAP of TAA10 and XX329 with three biological replicates were sampled, immediately frozen in liquid nitrogen and stored at − 80 °C.Each replicate contained three spikes and the grains in eight central spikelets of each spike were sampled. Sugar analysis was performed using ten grains in each sample, which were weighed and prepared for measurement. Soluble sugars of grain samples were extracted and dissolved in water, and the contents of sucrose, D-glucose and D-fructose in the aqueous extracts were enzymatically determined using a kit from R-Biopharm (Darmstadt, Germany) according to the manual (https://food.r-biopharm.com/wp-content/uploads/sites/2/2014/04/Roche_IFU_Sucrose-Glucose-fructose_EN_10716260035_2014-01.pdf). Statistical analysis of the difference in the sugar content between TAA10 and XX329 was performed using Student’s *t*-test.

### RNA extraction and transcriptome sequencing

Total RNA was extracted from grains at 6 DAP of TAA10 and XX329 with three biological replicates using TRIzol reagent (Invitrogen), according to the manual. RNA concentration was measured using a NanoDrop 2000 spectrophotometer (Thermo Fisher Scientific, Inc., USA) and RNA integrity was assessed using an Agilent 2100 Bioanalyzer (Agilent Technologies, Inc., USA). Non-stranded paired-end sequencing libraries with an average insert size of 400 bp were prepared with TruSeq RNA Sample Preparation Kit v2 (Illumina, USA) and sequenced using HiSeq 2000 (Illumina, USA) based on the manufacturer’s instructions. Raw data obtained from Illumina sequencing were processed and filtered using the Illumina pipeline (http://www.illumina.com) to generate FastQ files. Low quality reads were filtered with the following parameters: removing the reads containing adaptor sequences, more than 3% ambiguous bases (noted as N) and 50% low quality bases (Phred quality score Q < 30). Finally, approximately 6 Gb high-quality 125-bp paired-end reads were generated from each library. The RNA-Seq reads used for this study were deposited at the National Center for Biotechnology Information Short Read Archive (http://www.ncbi.nlm.nih.gov/sra/) under the accession number SRP117710.

### Alignment of RNA-Seq reads and expression analysis

The high-quality paired-end RNA-Seq reads from each library were aligned to the wheat reference genome (version TGACv1, http://plants.ensembl.org/Triticum_aestivum/) [32] using Hisat2 with parameters “--phred33 --max-intronlen 7000 -k 10 -t -p 4 --no-unal --ignore-quals --rdg 3,1 --rfg 3,1 --score-min L,0,-0.19”, and alignments with no more than one mismatch were retained [33]. Then, multiply mapped reads were filtered out by customized *Perl* script, and reads uniquely mapped to the annotated wheat reference genes were counted by HTSeq-count [34]. The uniquely mapped reads counts were used in the following FPKM (Fragments per Kilobase per Million mapped reads) calculation and differential expression analysis which were performed by edgeR package in R software [35]. Wheat reference genes with more than 2-fold changes and false discovery rate (FDR, Benjamini and Hochberg’s method)-adjusted *P*-value less than 0.05 were considered as differentially expressed genes.

### MapMan analysis

Wheat transcripts were annotated using the MapMan Mercator tool (http://mapman.gabipd.org /web/guest/mercator). The functional category analysis of differentially expressed genes was performed by MapMan version 3.6.0 [36]. Significantly overrepresented MapMan functional categories were determined by Fisher’s Exact Test (*P*-value < 0.05) and enrichment fold ≥1.5 compared with the whole genome background.

### Quantitative reverse transcription PCR (qRT-PCR) analysis

Total RNA (2 μg) from each sample was used to generate complementary DNA (cDNA) templates using HiScript Q RT SuperMix for qPCR (Vazyme Biotech, Ltd., China), according to the manufacturer’s instructions. QRT-PCR was performed using a SYBR Green PCR Master Mix (Vazyme Biotech, Ltd., China) on the CFX96 Real-Time PCR Detection System (Bio-Rad Laboratories, Inc., USA). The PCR conditions consisted of an initial step at 95 °C for 3 min followed by 40 cycles of 95 °C for 15 s, 60 °C for 15 s and 72 °C for 30 s. Specific primer pairs for qRT-PCR analysis are listed in Table S6. For each sample, the PCR analysis was independently repeated in triplicate, and the quantification of gene expression was carried out by the relative quantification method (2^-ΔΔCT^ method) with *β*-actin as an endogenous control [37].

## Results

### Dynamic comparison of developing grains between natural and resynthesized allohexaploid wheats

**Fig. 1:**
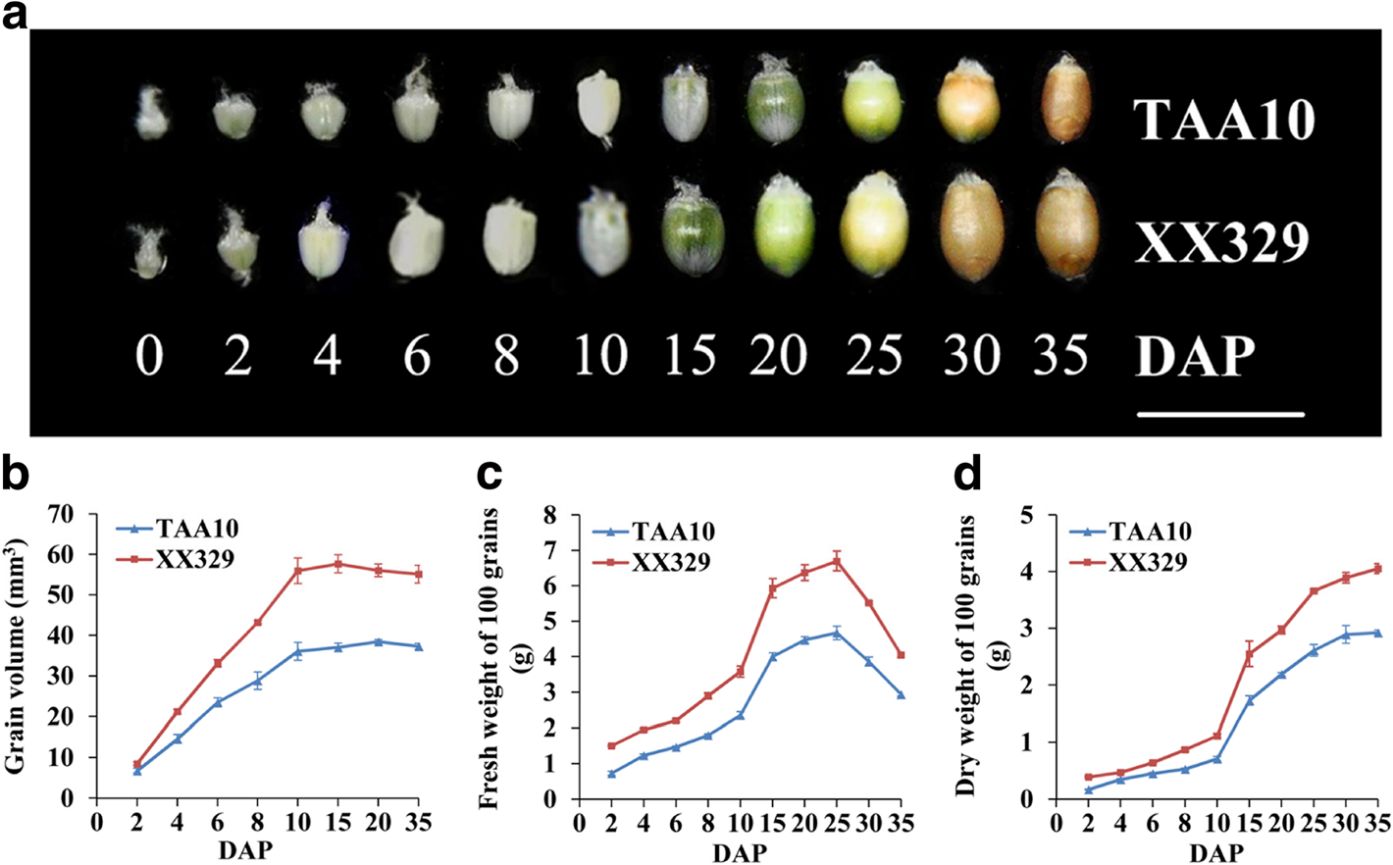
Dynamic comparison of grains at different developmental stages between the natural allohexaploid wheat TAA10 and the resynthesized allohexaploid wheat XX329. **a** Grain morphology at 0, 2, 4, 6, 8, 10, 15, 20, 25, 30 and 35 DAP of TAA10 and XX329. The scale bar represents 1 cm. **b** Grain volume at 2, 4, 6, 8, 10, 15, 20 and 35 DAP of TAA10 and XX329. The values are shown as means ± SE. **c**-**d** 100-grain fresh weight and dry weight at 2, 4, 6, 8, 10, 15, 20, 25, 30 and 35 DAP of TAA10 and XX329. The values are shown as means ± SE

To explore the underlying mechanism for the variation of grain size and weight between natural and resynthesized allohexaploid wheats (TAA10 and XX329), we firstly measured the grain volume of these two genotypes at 2, 4, 6, 8, 10, 15, 20 and 35 DAP. For each genotype, grain volume rapidly increased during the early stages of development and reached their maximal volume at about 10 DAP, which was consistent with previous studies [16]. When comparing these two genotypes, the grain volume of XX329 was significantly higher than that of TAA10 from 4 DAP and acquired the largest difference at 10 DAP. After that, the superiority of XX329 was maintained until all grains matured (Fig. 1b). Next, we measured the fresh and dry grain weight of TAA10 and XX329 at different stages of development. The fresh grain weight of TAA10 and XX329 consecutively increased from 2 DAP and acquired their maximal fresh weight at about 25 DAP, thereafter decreased until the grains matured (Fig. 1c). Different from the fresh grain weight, the dry grain weight of each genotype continued to increase until grain maturation at about 35 DAP (Fig. 1d). Notably, XX329 exhibited significantly higher fresh and dry grain weight than that of TAA10 at each stages of grain development, and the difference in dry grain weight between XX329 and TAA10 reached the maximum at about 35 DAP when all grains matured (Fig. 1c-d).

**Fig. 2:**
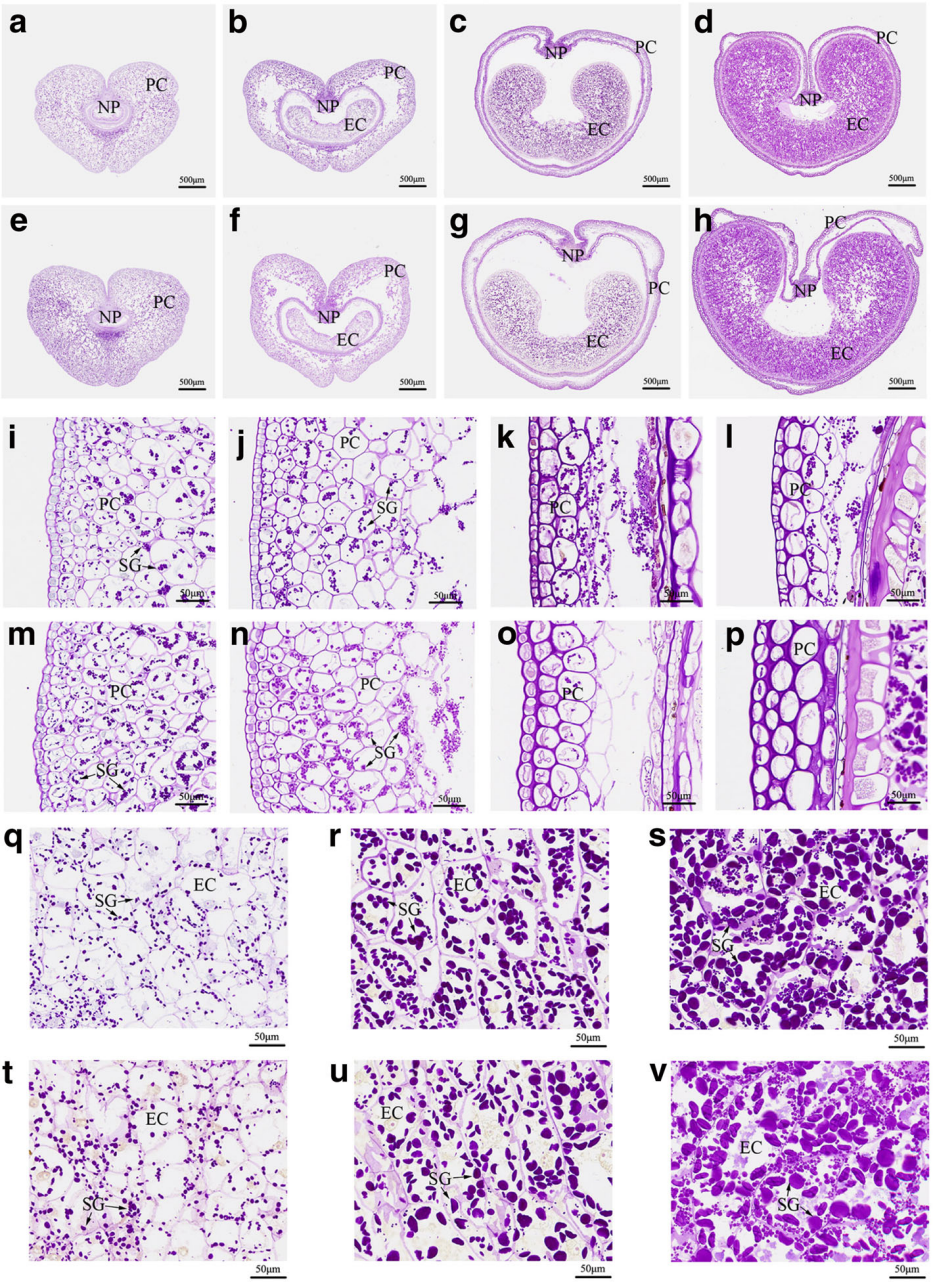
Cytological observations by cross-sections of grains at different developmental stages between the natural allohexaploid wheat TAA10 and the resynthesized allohexaploid wheat XX329. **a**-**d** Cross-sections of grains at 2 (**a**), 6 (**b**), 10 (**c**) and 15 (**d**) DAP in TAA10.The scale bars represent 500 μm. **e**-**h** Cross-sections of grains at 2 (**e**), 6 (**f**), 10 (**g**) and 15 (**h**) DAP in XX329. The scale bars represent 500 μm. **i**-**l** The pericarp cells of grains at 2 (**i**), 6 (**j**), 10 (**k**) and 15 (**l**) DAP in TAA10. The scale bars represent 50 μm. **m**-**p** The pericarp cells of grains at 2 (**m**), 6 (**n**), 10 (**o**) and 15 (**p**) DAP in XX329. The scale bars represent 50 μm. **q**-**s** The endosperm cells of grains at 6 (**q**), 10 (**r**) and 15 (**s**) DAP in TAA10. The scale bars represent 50 μm. **t**-**v** The endosperm cells of grains at 6 (**t**), 10 (**u**) and 15 (**v**) DAP in XX329. The scale bars represent 50 μm. NP, nucellar projection; PC, pericarp cell; EC, endosperm cell; SG, starch granule

**Fig. 3:**
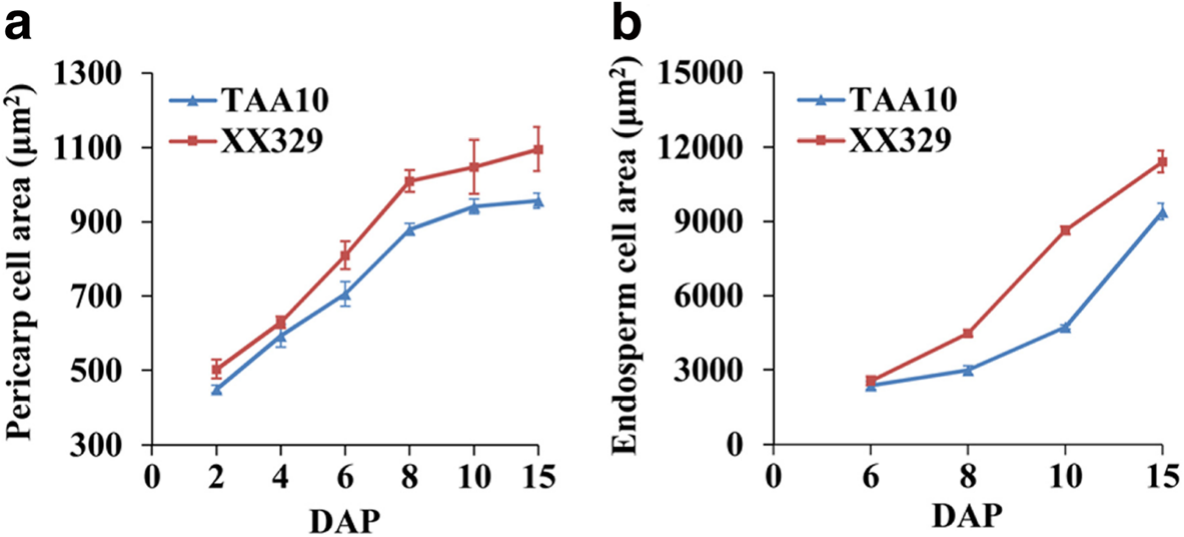
Cell area of the pericarp and endosperm during the early stages of grain development between the natural allohexaploid wheat TAA10 and the resynthesized allohexaploid wheat XX329. **a** Cell area of the pericarp at 2, 4, 6, 8, 10 and 15 DAP of TAA10 and XX329. The values are shown as means ± SE. **b** Cell area of the endosperm at 6, 8, 10 and 15 DAP of TAA10 and XX329. The values are shown as means ± SE

The development of maternal grain tissue is mediated by regulated cell expansion and coordinated with endosperm growth [38]. Thus, we decided to measure and compare the cell size of the pericarp and endosperm in TAA10 and XX329 during the early stages of grain development (2, 4, 6, 8, 10 and 15 DAP) by cytological observation. The pericarp cell area of each genotype increased by 2 fold from 2 to 15 DAP and the growth rate of pericarp cells at 2-8 DAP was faster than that at 8-15 DAP. Moreover, the pericarp cells of XX329 exhibited faster growth rate and greater cell area than those of TAA10 during the early stages of grain development (Fig. 2i-p; Fig. 3a). Correspondingly, the growth of the endosperm cells kept pace with that of the pericarp cells. The endosperm cell size of each genotype increased by 3-4 folds from 6 to 15 DAP. Moreover, XX329 exhibited much greater endosperm cell area than that of TAA10 from 8 to 15 DAP (Fig. 2q-v; Fig. 3b). Notably, we found that the cellularization of the endosperm was complete and the starch granules were initially formed in quantity in the endosperm cells by 6 DAP (Fig. 2). Taken together, grains at 6 DAP were selected for further comparative transcriptome analysis.

### Comparative transcriptome profile of 6-DAP grains between natural and resynthesized allohexaploid wheats

**Fig. 4:**
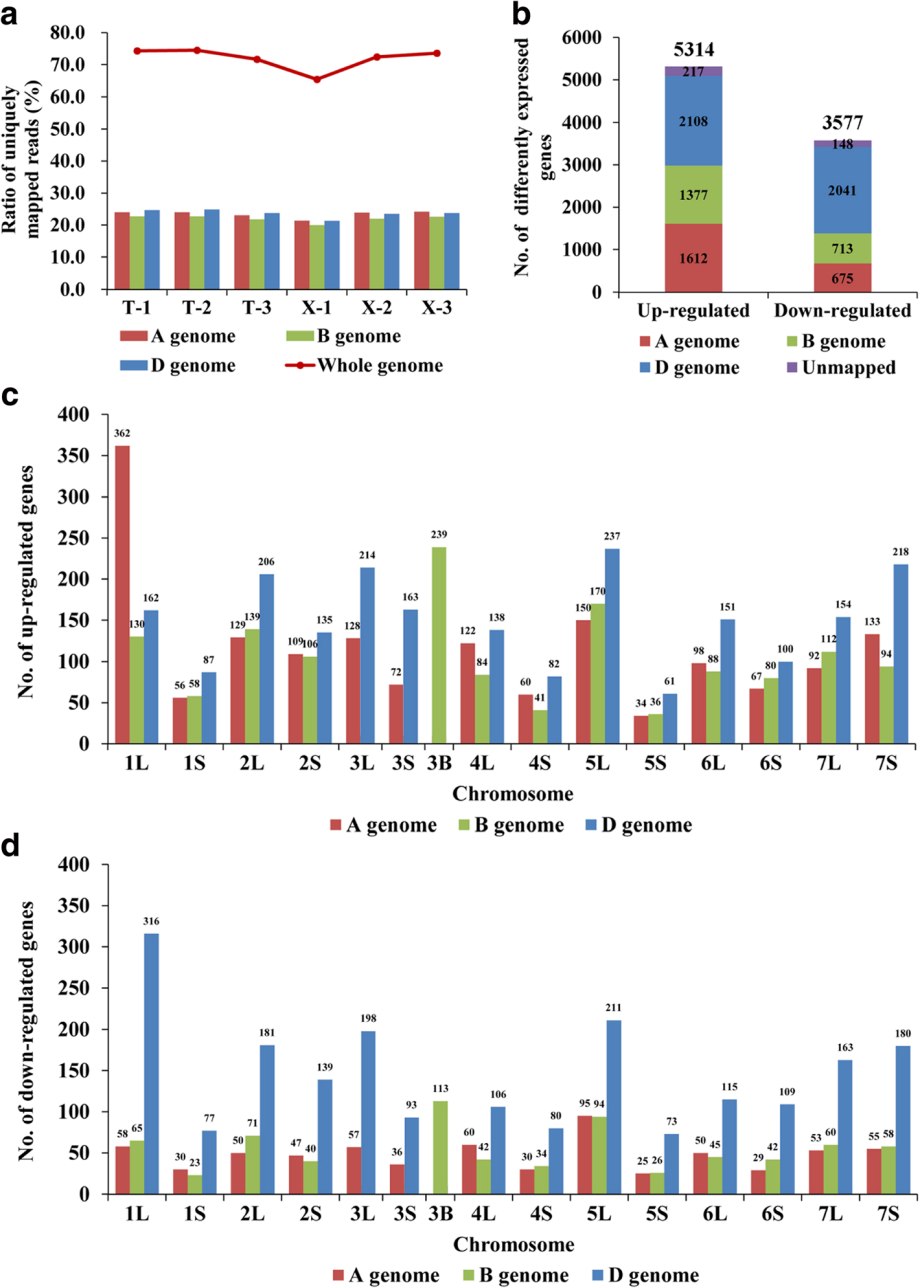
Transcriptome sequencing reads mapping and differentially expressed analysis. **a** The ratio of the uniquely mapped reads in each biological replicate of TAA10 and XX329. **b** The numbers of up-regulated and down-regulated genes on the A, B and D genomes in the resynthesized allohexaploid wheat XX329 relative to the natural allohexaploid wheat TAA10. (c-d) The numbers of up-regulated (**c**) and down-regulated genes (**d**) in XX329 relative to TAA10 on the long and short arms of different chromosomes. There is no separation for long and short arms on the chromosome 3B

To dissect the potential molecular basis for the difference in grain size between natural and resynthesized allohexaploid wheats, we performed RNA sequencing of 6-DAP grains of TAA10 and XX329 with three biological replicates. After removal of low-quality sequencing reads, a total of approximately 162.2 million 125 bp paired-end reads were generated, with an average of 27.0 million filtered reads for each library. About 65.4%-74.5% of the high-quality reads were uniquely mapped to wheat reference genome sequence. The ratios of uniquely mapped reads for TAA10 and XX329 on the A, B and D genomes of allohexaploid wheat ranged from 21.5 to 23.7%, indicating that these reads were equally distributed among the three subgenomes (Fig. 4a).

**Fig. 5:**
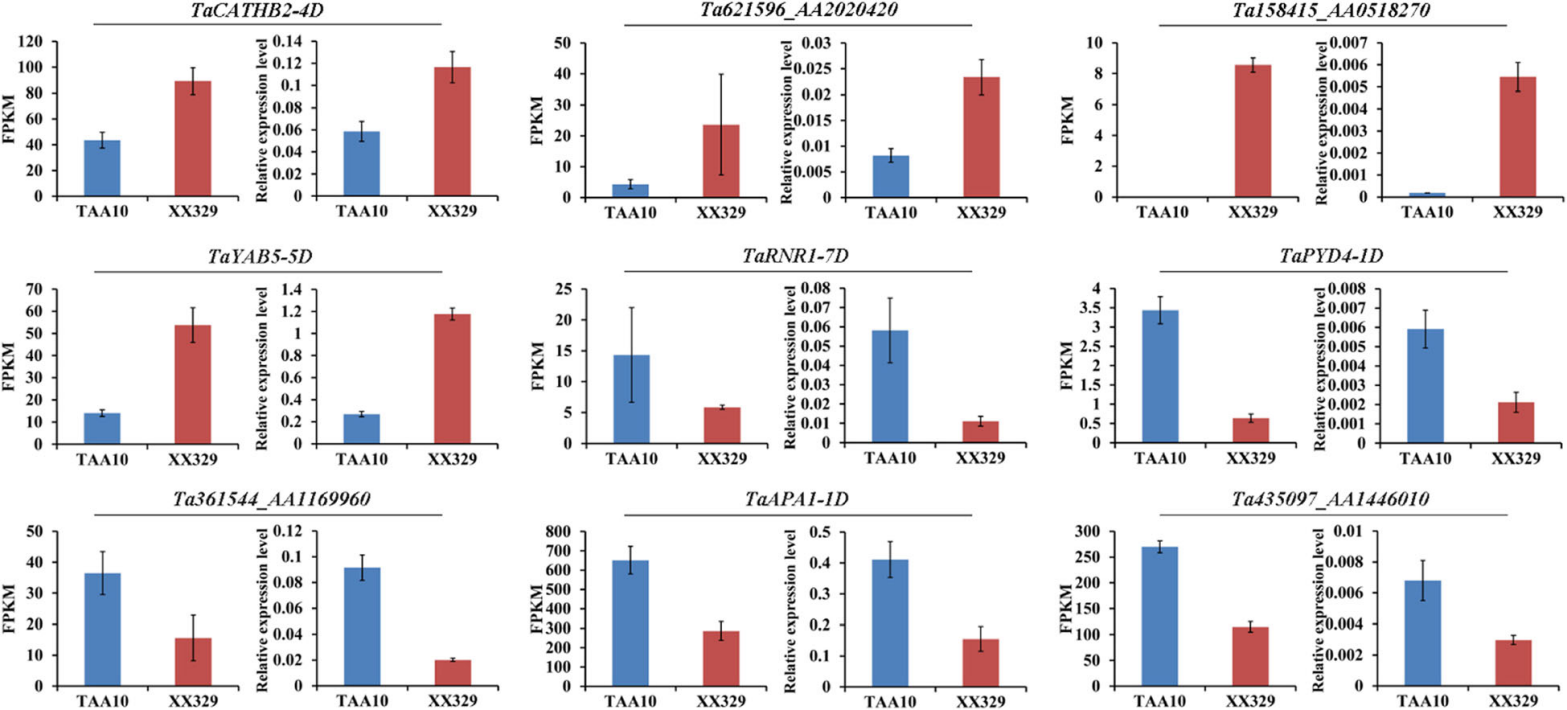
Verification of 9 differentially expressed genes by qRT-PCR. The values are shown as means ± SD

To identify the differentially expressed genes between 6-DAP grains of the natural allohexaploid TAA10 and resynthesized allohexaploid wheat XX329, we performed the expression analysis using the uniquely mapped reads for each library. The correlation coefficients of different biological replicates ranged from 0.972 to 0.986 (Additional file 1: Figure S1). In total, 69,711 TGACv1 reference genes were expressed (FPKM ≥0.5 and Reads Counts ≥10) among the two genotypes and the number in TAA10 and XX329 was 69,162 and 69,195, respectively. Of 69,711 genes, 8891 (12.75%) were differentially expressed genes between TAA10 and XX329 (fold change ≥2 and false discovery rate (FDR) adjusted *P* < 0.05) (Additional file 2: Table S1). Compared to TAA10, the number of up-regulated genes in XX329 (5314) was much higher than that of down-regulated genes (3577) (Fig. 4b). To validate the RNA-Seq results, qRT-PCR was performed for 9 randomly selected differentially expressed genes. As shown in Fig. 5, the fold changes in gene expression determined by qRT-PCR were consistent with the changes of normalized expression level (FPKM) determined by RNA-Seq. The detailed information for these genes was shown in Additional file 3: Table S6.

According to the wheat genome annotation (version TGACv1, http://plants.ensembl.org/Triticum_aestivum/), 8526 (95.89%) differentially expressed genes could be assigned to chromosomes, and the numbers on the A, B and D genomes were 2287, 2090 and 4149, respectively (Fig. 4b). Notably, the number of down-regulated genes in XX329 relative to TAA10 on each chromosome of the D genome was higher than that on each corresponding chromosome of the A and B genomes. In addition, we found that down-regulated genes were enriched on chromosome 1DL, whereas up-regulated genes were overrepresented on chromosome 1AL (Fig. 4c-d).

### Functional annotation and categorization of the differentially expressed genes

The MapMan analysis was performed to analyze the functional annotation and categorization of the differentially expressed genes. In total, 7039 of 8891 differentially expressed genes between TAA10 and XX329 could be assigned to the MapMan categories, of which 2022 were “not assigned” to a functional category. The number of genes were assigned to the functional category “RNA” was highest (842), followed by “protein” (815), “misc.” (811) and “signaling” (397) (Table 1; Additional file 4: Table S2). Furthermore, the functional enrichment analysis of differentially expressed genes revealed that 6 primary functional categories were significantly overrepresented, including “misc.”, “cell wall”, “secondary metabolism” “major CHO (carbohydrate) metabolism”, “hormone metabolism” and “gluconeogenese/ glyoxylate cycle” (Table 1). In addition, enriched functional subcategories in each overrepresented category were further investigated. The results exhibited the numbers of enriched secondary functional categories in “misc.”, “cell wall”, “secondary metabolism”, “major CHO (carbohydrate) metabolism”, “hormone metabolism” and “gluconeogenese/ glyoxylate cycle” were 10, 4, 4, 2, 2 and 1, respectively (Additional file 5: Table S3). Although the precise roles of these differentially expressed genes are unknown, the alteration of genes may contribute to the observed variation in grain size between TAA10 and XX329.

**Table 1:** Numbers of differentially expressed genes and enrichment significance in 35 MapMan primary functional categories

Primary functional category	No. of differentially expressed genes	*P*-value
1 PS	31	0.992
2 major CHO (carbohydrate) metabolism	73	7.92E-11
3 minor CHO metabolism	37	0.232
4 glycolysis	8	0.858
5 fermentation	7	0.403
6 gluconeogenese/glyoxylate cycle	6	0.020
7 OPP	3	0.950
8 TCA / org. Transformation	10	0.993
9 mitochondrial electron transport / ATP synthesis	11	0.999
10 cell wall	201	1.54E-14
11 lipid metabolism	86	0.922
12 N-metabolism	5	0.773
13 amino acid metabolism	72	0.407
14 S-assimilation	1	0.871
15 metal handling	11	0.889
16 secondary metabolism	221	1.60E-12
17 hormone metabolism	226	3.66E-06
18 Co-factor and vitamine metabolism	2	0.999
19 tetrapyrrole synthesis	1	0.999
20 stress	394	0.256
21 redox	28	0.999
22 polyamine metabolism	3	0.752
23 nucleotide metabolism	28	0.957
24 Biodegradation of Xenobiotics	27	0.052
25 C1-metabolism	5	0.831
26 misc	811	1.96E-29
27 RNA	842	0.647
28 DNA	158	0.999
29 protein	815	0.999
30 signalling	397	0.999
32 μ RNA, natural antisense etc.	0	0.962
31 cell	179	0.999
33 development	222	0.893
34 transport	303	0.960
35 not assigned	2022	0.004

**Fig. 6:**
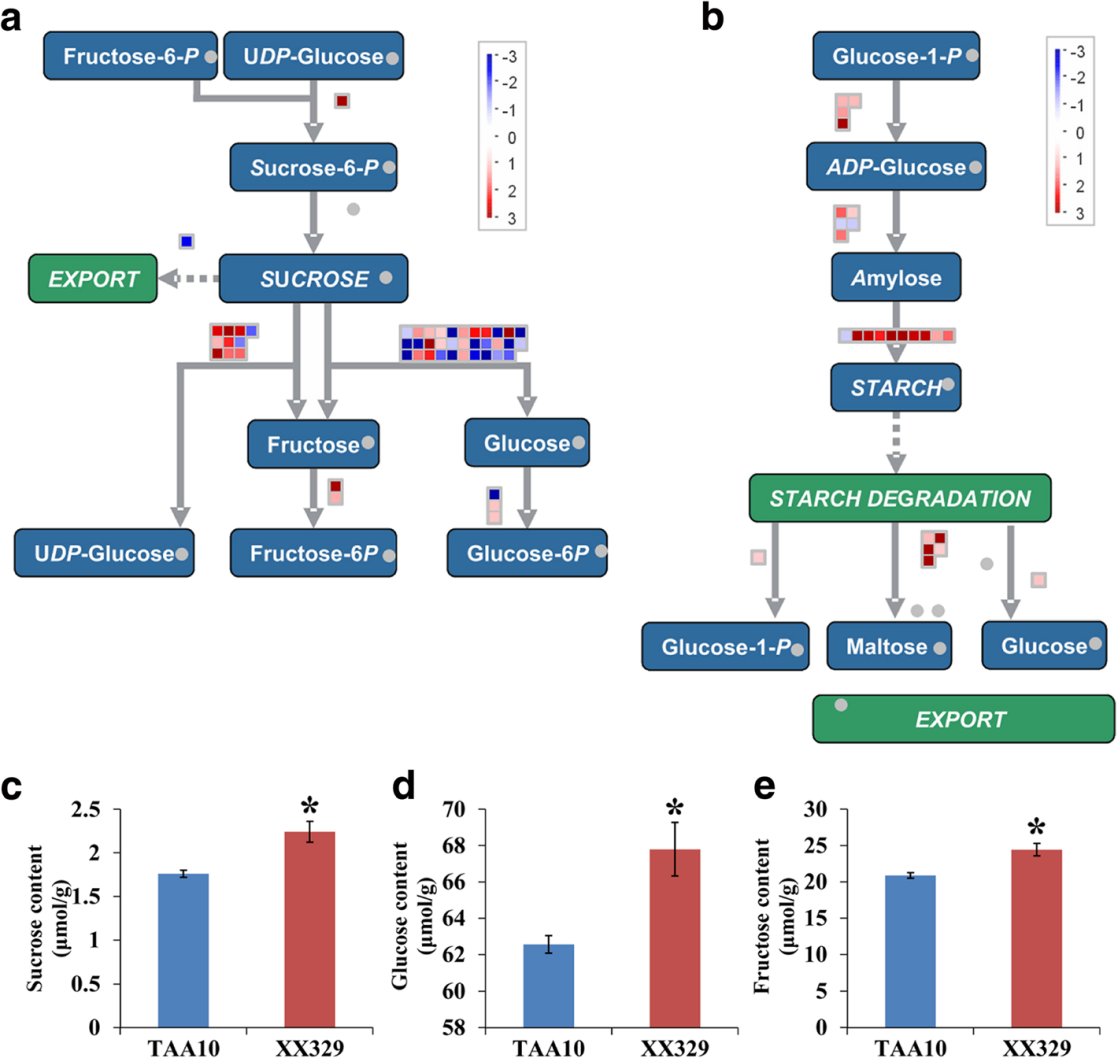
MapMan pathways in sucrose-starch metabolism and sugar content analysis. **a**-**b** Differentially expressed genes between TAA10 and XX329 in sucrose degradation (**a**) and starch synthesis (**b**) pathways. Individual genes are represented by small squares. The colour of squares represents the value of log_2_ fold change. Red represents up-regulation and blue represents down-regulation in XX329 relative to TAA10. **c**-**e** Sucrose (**c**), glucose (**d**) and fructose (**e**) contents in grains at 6 DAP of TAA10 and XX329. The values are shown as means ± SE. * indicates the significance at the 0.05 level (Student’s *t* test)

Carbohydrate metabolism is defined as the various biochemical processes responsible for the formation, breakdown and interconversion of carbohydrates. In wheat developing grains, sucrose metabolism is important for providing material and energy source to the biosynthesis of cell wall and starch. Our MapMan analysis revealed that differentially expressed genes involved in sucrose degradation exhibited significantly enrichment, including sucrose synthase (SUS) and invertase genes. Eight of 10 differentially expressed genes encoding sucrose synthase were up-regulated in XX329 relative to TAA10. Of 29 differentially expressed genes encoding cell wall (13), vacuolar (15) and neutral (1) invertases, the number of up-regulated genes in XX329 relative to TAA10 was 7, 7 and 0, respectively (Table 2; Fig. 6a). Consistently, XX329 accumulated much higher contents of sucrose, D-glucose and D-fructose than TAA10 (Fig. 6c-e). In addition, our MapMan analysis revealed that 21 differentially expressed genes were enriched in the starch biosynthesis pathway, which encoded four key enzymes, that is starch branching enzyme (SBE), ADP-glucose pyrophosphorylase (AGPase), starch synthases (SS) and starch debranching enzyme (DBE). All differentially expressed genes encoding the AGPase plastidial large subunit (4) and DBE (2) including Isoamylase 1 (1) and Isoamylase 2 (1) were significantly up-regulated in XX329 compared to TAA10. Of 10 differentially expressed *SBE* genes, 9 *SBE2* genes were up-regulated, whereas the other one *SBE1* gene was down-regulated in XX329 relative to TAA10. Three of 5 differentially expressed *SS* genes were up-regulated in XX329 compared to TAA10 (Table 2; Fig. 6b). To further validate the accuracy of RNA-seq results, qRT-PCR was performed for 5 randomly selected differently expressed genes involved in carbohydrate metabolism, among which the expression changes of 4 genes were consistent with the RNA-seq results (Additional file 6: Figure S2).

Grain growth during early development involved in the cell expansion of the pericarp required a mass of cell wall biosynthesis. Interestingly, we found that 201 differentially expressed genes were enriched in the primary functional category “cell wall”, among which 4 functional subcategories exhibited significant overrepresentation, including “cellulose synthesis”, “degradation”, “modification” and “cell wall proteins” (Additional file 5: Table S3). For the subcategory of cellulose synthesis, 26 differentially expressed genes were significantly overrepresented, mainly including *cellulose synthase* and *COBRA* genes (12 and 8), and the number of up-regulated genes encoding cellulose synthase and COBRA in XX329 relative to TAA10 was 5 and 6, respectively. Moreover, we identified 57 differentially expressed genes involved in cell wall degradation, including 22 up-regulated and 35 down-regulated genes, such as cellulase and pectinase genes. Notably, of differentially expressed genes encoding expansin (26) and Xyloglucan transglucosylase/hydrolase (XTH) (31) associated with cell wall modification, the number of up-regulated genes in XX329 relative to TAA10 was 16 and 18, respectively. In addition, of 24 differentially expressed genes enriched in the functional subcategory “cell wall proteins”, all five genes encoding arabinogalactan protein (AGP) were up-regulated in XX329 compared to TAA10, whereas only 7 of 15 genes encoding Leucine-rich repeat (LRR) family protein were up-regulated (Additional file 7: Table S4).

**Table 2:** The differentially expressed genes involved in sucrose degradation and starch synthesis pathways

Functional category	Gene ID^a^	Description	Log_2_ fold change^b^	FDR-adjusted *P*-value
Major CHO metabolism. Degradation. sucrose. Invertases	TRIAE_CS42_4AL_TGACv1_288169_AA0939320	Vacuolar invertase	1.42	7.74E-06
	TRIAE_CS42_4AL_TGACv1_288169_AA0939350	Vacuolar invertase	2.27	5.23E-04
	TRIAE_CS42_4DL_TGACv1_342608_AA1117890	Cell wall invertase	1.56	2.30E-02
	TRIAE_CS42_5BL_TGACv1_404129_AA1285640	Cell wall invertase	6.21	4.38E-21
	TRIAE_CS42_5DL_TGACv1_433511_AA1415210	Cell wall invertase	2.29	8.19E-06
	TRIAE_CS42_6AS_TGACv1_487805_AA1573040	Cell wall invertase	1.81	1.56E-09
	TRIAE_CS42_6BS_TGACv1_514515_AA1660840	Cell wall invertase	1.24	2.21E-09
	TRIAE_CS42_7AS_TGACv1_569119_AA1807690	Vacuolar invertase	2.38	5.75E-21
	TRIAE_CS42_7AS_TGACv1_569629_AA1820610	Vacuolar invertase	1.49	1.16E-02
	TRIAE_CS42_7AS_TGACv1_571086_AA1844330	Vacuolar invertase	1.26	3.85E-04
	TRIAE_CS42_7DS_TGACv1_622623_AA2042790	Vacuolar invertase	1.42	9.07E-06
	**TRIAE_CS42_7DS_TGACv1_622976_AA2048120**	Vacuolar invertase	3.26	4.97E-28
	TRIAE_CS42_U_TGACv1_645154_AA2143350	Cell wall invertase	1.05	1.71E-03
	TRIAE_CS42_U_TGACv1_645154_AA2143370	Cell wall invertase	1.03	2.48E-07
	TRIAE_CS42_1AL_TGACv1_002147_AA0039110	Cell wall invertase	−3.03	1.42E-07
	TRIAE_CS42_1BL_TGACv1_030243_AA0083360	Cell wall invertase	−3.29	5.86E-07
	TRIAE_CS42_1DL_TGACv1_061368_AA0193220	Cell wall invertase	−2.93	5.66E-05
	TRIAE_CS42_2AL_TGACv1_093126_AA0272730	Cell wall invertase	−1.95	1.27E-04
	TRIAE_CS42_2BL_TGACv1_130262_AA0407610	Cell wall invertase	−3.38	6.12E-09
	TRIAE_CS42_2DL_TGACv1_159903_AA0544110	Cell wall invertase	−1.14	4.71E-04
	TRIAE_CS42_4AL_TGACv1_288169_AA0939300	Vacuolar invertase	−4.23	6.36E-07
	TRIAE_CS42_6AS_TGACv1_486383_AA1560850	Vacuolar invertase	−3.18	4.03E-06
	TRIAE_CS42_6BS_TGACv1_513852_AA1650710	Vacuolar invertase	−1.89	4.46E-02
	TRIAE_CS42_6BS_TGACv1_515140_AA1667640	Vacuolar invertase	−3.17	9.25E-05
	TRIAE_CS42_6DS_TGACv1_542882_AA1732150	Vacuolar invertase	−2.92	3.76E-07
	TRIAE_CS42_7AS_TGACv1_570692_AA1839380	Vacuolar invertase	−2.64	2.06E-05
	TRIAE_CS42_7DS_TGACv1_621614_AA2021150	Vacuolar invertase	−1.54	8.16E-03
	TRIAE_CS42_7DS_TGACv1_622753_AA2044760	Vacuolar invertase	−4.78	1.20E-19
	TRIAE_CS42_U_TGACv1_642336_AA2115990	Neutral invertase	−1.11	8.73E-03
Major CHO metabolism. Degradation. sucrose. Susy	TRIAE_CS42_1AL_TGACv1_002916_AA0046300	Sucrose synthase	4.20	1.42E-09
	TRIAE_CS42_2AS_TGACv1_112101_AA0330380	Sucrose synthase	2.25	2.76E-16
	TRIAE_CS42_2BS_TGACv1_145971_AA0451230	Sucrose synthase	1.85	1.34E-11
	TRIAE_CS42_2DL_TGACv1_159703_AA0541730	Sucrose synthase	1.89	5.80E-05
	TRIAE_CS42_2DS_TGACv1_177457_AA0577630	Sucrose synthase	2.71	9.79E-17
	TRIAE_CS42_4AL_TGACv1_288793_AA0958320	Sucrose synthase	1.30	1.73E-05
	TRIAE_CS42_7AS_TGACv1_569135_AA1808380	Sucrose synthase	2.60	1.96E-16
	TRIAE_CS42_7DS_TGACv1_622658_AA2043420	Sucrose synthase	2.94	1.09E-24
	**TRIAE_CS42_6DL_TGACv1_527850_AA1709240**	Sucrose synthase	−1.74	3.14E-07
	TRIAE_CS42_7DL_TGACv1_604951_AA2003310	Sucrose synthase	−1.96	3.18E-09
Major CHO metabolism. Synthesis. starch. AGPase	TRIAE_CS42_1AL_TGACv1_000939_AA0022300	AGPase plastidial large subunit	9.51	9.06E-110
	TRIAE_CS42_1BL_TGACv1_031712_AA0119450	AGPase plastidial large subunit	1.26	9.23E-05
	TRIAE_CS42_7AS_TGACv1_569682_AA1821750	AGPase plastidial large subunit	1.29	2.65E-06
	**TRIAE_CS42_7DS_TGACv1_622617_AA2042690**	AGPase plastidial large subunit	1.52	6.37E-08
Major CHO metabolism. Synthesis. starch. Debranching	TRIAE_CS42_1DL_TGACv1_061778_AA0203340	Isoamylase 2	1.15	3.90E-07
	TRIAE_CS42_7DS_TGACv1_622495_AA2040790	Isoamylase 1	1.07	6.13E-04
Major CHO metabolism. Synthesis. starch. Starch branching	TRIAE_CS42_2AL_TGACv1_095785_AA0314520	Starch branching enzyme 2	2.85	4.50E-10
	TRIAE_CS42_2BL_TGACv1_132845_AA0440100	Starch branching enzyme 2	1.43	3.85E-05
	**TRIAE_CS42_2DL_TGACv1_158200_AA0512220**	Starch branching enzyme 2	1.87	1.96E-09
	TRIAE_CS42_7AL_TGACv1_556597_AA1766610	Starch branching enzyme 2	2.83	1.68E-14
	TRIAE_CS42_7AL_TGACv1_556597_AA1766630	Starch branching enzyme 2	2.97	3.57E-14
	TRIAE_CS42_7BL_TGACv1_577598_AA1879190	Starch branching enzyme 2	4.12	3.24E-14
	TRIAE_CS42_7BL_TGACv1_580343_AA1913520	Starch branching enzyme 2	2.28	6.22E-08
	TRIAE_CS42_7DL_TGACv1_603128_AA1976400	Starch branching enzyme 2	2.77	2.09E-05
	TRIAE_CS42_7DL_TGACv1_603128_AA1976410	Starch branching enzyme 2	4.79	9.97E-23
	TRIAE_CS42_7AL_TGACv1_556924_AA1773500	Starch branching enzyme 1	−1.06	2.63E-02
Major CHO metabolism. Synthesis. starch.starch synthase	TRIAE_CS42_1AS_TGACv1_020151_AA0075220	Starch synthase 3	1.77	3.39E-12
	TRIAE_CS42_1BL_TGACv1_030553_AA0093880	Starch synthase	1.93	1.18E-10
	TRIAE_CS42_1BS_TGACv1_050983_AA0177050	Starch synthase 3	1.07	2.87E-05
	TRIAE_CS42_2BL_TGACv1_131141_AA0423210	Starch synthase 3	−1.09	9.71E-04
	**TRIAE_CS42_2DL_TGACv1_157939_AA0502660**	Starch synthase	−1.02	1.31E-03

^a^Genes for qRT-PCR analysis are shown in bold

^b^Fold change indicates that gene expression change in XX329 compared to TAA10

Based on the MapMan analysis, we found that 226 differentially expressed genes were enriched in the primary functional category “hormone metabolism”, including auxin, abscisic acid, ethylene and gibberellins metabolism (Additional file 5: Table S3).. Of 10 differentially expressed genes involved in auxin metabolism, 8 genes encoded the IAA-leucine-resistant (ILR) proteins regulating auxin homeostasis were up-regulated in XX329 compared to TAA10. Moreover, 7 genes involved in signal transduction of abscisic acid, such as *ABI3* and *FUS3*, were also significantly up-regulated in XX329 compared to TAA10. Notably, we identified that a large number of differentially expressed genes (108) were associated with the ethylene metabolism, which were involved in the ethylene biosynthesis, degradation and signal transduction. In addition, the expression of 9 GA20ox genes involved in gibberellins biosynthesis were significantly altered, among which 6 were up-regulated and 3 were down-regulated in XX329 relative to TAA10 (Additional file 8: Table S5).

## Discussion

### Genetic variation of D genome induced drastic changes of gene expression in allohexaploid wheats

*Aegilops tauschii*, the D-genome progenitor of *Triticum aestivum*, encompasses extensive variation for various traits of potential economic importance, including yield, biotic and abiotic stress tolerance, quality and nutrition [21–24, 39, 40]. Until now, most studies focused on understanding the genetic and morphological diversity of *Ae. tauschii* germplasm and their functions under a allohexaploid genetic background, but little is known about their effects on gene expression [41–43]. The natural and resynthesized allohexaploid wheats with near-identical A and B genomes could provide a good example for investigating the effects of different D genomes on gene expression during allopolyploid wheat formation and evolution [44]. Recently, it was reported that the genetic variability of the progenitor of the D genome (*Ae. tauschii*) does not lead to significant changes in gene expression in leaves of resynthesized allohexaploid wheats, and similar features were observed by direct comparison between natural and resynthesized allohexaploid wheats [30]. In the present study, we found that 8891 genes were differentially expressed between 6-DAP grains of natural and resynthesized allohexaploid wheats with near-identical AB genomes, which is much higher than that in leaves of previous studies [30], suggesting that the genetic variation of the D genome induced drastic alterations of gene expression in grains of allohexaploid wheats. Interestingly, the number of down-regulated genes on the D genome of resynthesized relative to natural allohexaploid wheat (2041) is much higher than that on A and B genomes (675 and 713), which may be due to modifications of D genome to the genomic shocks that occurred during and after allopolyploidization [45]. It should to be noted that the number of up-regulated genes on the AB genome in resynthesized relative to natural allohexaploid wheat (2989) was significantly higher than that of down-regulated genes (1388), suggesting that the D genome of *Aegilops tauschii* mainly contributed to positive effects on gene expression of AB genomes by inter-genomic interactions in resynthesized allohexaploid wheats.

Accumulated data revealed that the repression of one homologous gene is compensated by the activation of the others in allopolyploid wheat [44, 46, 47]. For example, a recent study of leaf gene expression in bread wheat identified many small regions of genome dominance (transcripts of homoeologues from one genome were more abundant than the others) and many larger regions of genome repression (transcripts of homoeologues from one genome were less abundant than the others) [47]. Interestingly, we found that the genomic region on chromosome 1DL enriched down-regulated genes in resynthesized relative to the natural allohexaploid wheat. In contrast, up-regulated genes in resynthesized allohexaploid wheat were overrepresented on the corresponding homoeologous region of chromosome 1AL. Taken together, these data provide further evidence for the dominance/repression of gene expression on specific genomic regions in resynthesized allohexaploid wheat, which may play an important role in homoeologous genome divergence and contribute to genomic asymmetry. However, this feature is not observed on chromosome 1BL, which is needed for further investigation.

### Alteration of sucrose metabolism was essential for the formation of large grains in resynthesized allohexaploid wheats

Grain development is highly dependent on the metabolic utilization of sucrose, which can provide carbon for the synthesis of cell wall polysaccharides and starch [16, 48]. In this study, we found that the expression level of 26 genes involved in sucrose degradation in 6-DAP grains of the resynthesized allohexaploid wheat was significantly higher than those of the natural allohexaploid wheat. Sucrose synthase is widely believed to be the main route of entry of carbon from sucrose into cellular metabolism in plants, which catalyzed the reaction of sucrose to UDP-glucose and fructose [49]. In wheat, genetic studies exhibited that sucrose synthase 1 and 2 genes (*TaSus1 and TaSus2*) are associated with TGW [50]. Interestingly, 8 of 10 (80%) genes encoding sucrose synthase were up-regulated in resynthesized relative to natural allohexaploid wheat. On the other hand, sucrose is also a signal molecule in regulating gene expression and normal grain development in diverse cell types [51, 52]. In general, sucrose favors differentiation and maturation, whereas hexoses favor cell division and expansion [53, 54]. Consistent with this notion, the resynthesized allohexaploid wheat showed higher contents of glucose and fructose in grains at 6 DAP than those of the natural allohexaploid wheat. Collectively, these data indicated that the up-regulation of sucrose synthase genes in resynthesized allohexaploid wheat could accelerate the process of cell growth compared to natural allohexaploid wheat, which plays important roles in the differences in grain size and weight between the natural and resynthesized allohexaploid wheats.

### Rate of cell expansion contributed to the variation in grain size between resynthesized and natural allohexaploid wheats

Grain morphogenesis is dependent on the regulation of cell division and expansion [55]. Cell proliferation is almost completed by 2 DAP in the pericarp. Later, filial grain organs grow and expand rapidly within the maternal pericarp. Thereby, the growth of the endosperm must be coordinated with the maternal pericarp development, which collectively affects the grain development [38]. In the present study, we found that grains of natural and resynthesized allohexaploid wheats reach their maximal volume at about 10 DAP, whereas the grain size of the resynthesized allohexaploid wheat is significantly larger than that of the natural allohexaploid wheat. In accordance with the difference of grain size, the cell areas and growth rates of the pericarp and endosperm in the resynthesized allohexaploid wheat are higher than those in the natural allohexaploid wheat during the early stages of grain development. Therefore, the observed variation in grain size between natural and resynthesized allohexaploid wheats could be traced back to the different growth rate of the filial tissues.

Cell growth requires the cell wall to be irreversibly stretched through a wall loosening process, followed by the deposition of new wall material [56]. An important observation of this study is that the differentially expressed genes between natural and resynthesized allohexaploid wheats were significantly enriched in cell wall metabolism, which was well correlated with the difference in grain size between natural and resynthesized allohexaploid wheats at the critical period of early grain expansion (6 DAP). For example, expansins are the only proteins to directly induce cell wall extension by disrupting the hydrogen bonds between cellulose microfibrils and crosslinking glycans and permitting turgor-driven cell wall extension [57, 58]. In wheat, expansins are shown directly to induce cell wall extension, and expression of expansin genes is associated with grain size [59]. Interestingly, 16 of 26 (61.5%) differentially expressed expansins genes were up-regulated in resynthesized relative to natural allohexaploid wheat*.* Genes of the *COBRA* family are involved in various types of cell expansion and cell wall biosynthesis [60]. Of 8 differentially expressed COBRA-like genes, 6 (75%) were up-regulated in resynthesized relative to natural allohexaploid wheat. In expanding plant cells, the cellulose–xyloglucan network is considered the main load-bearing network that controls the extent of cell expansion [61]. *XTH* genes are important wall-loosening genes whose function in wall remodelling is to cleave xyloglucan to incorporate new molecules and catalyse linkage between xyloglucan and cellulose [62, 63]. We found that 18 of 31 (58.1%) genes encoding XTH proteins were up-regulated in resynthesized relative to natural allohexaploid wheat. However, different from the expression patterns of *XTH* genes, the majority of differentially expressed cellulose synthase genes (7/12, 58.3%) were down-regulated in resynthesized relative to natural allohexaploid wheat. Taken together, we proposed that these differentially expressed genes may contribute to the variation in grain size and weight between resynthesized and natural allohexaploid wheats.

### Comparison of differentially expressed genes with previously known QTL and candidate genes controlling wheat grain size and weight

Recently, we identified three QTL regions associated with grain size and shape on chromosomes 2DS, 2DL and 7DS, respectively, using the F_2_ and F_2:3_ populations derived from the natural allohexaploid wheat TAA10 and resynthesized allohexaploid wheat XX329 [64]. In the present study, comparative transcriptome analysis at 6-DAP grains between TAA10 and XX329 were performed, which may provide useful information for discovering the candidate genes underpinning phenotypic variation [65]. Comparative analysis revealed that the numbers of differentially expressed genes located within the QTL regions on chromosomes 2DS, 2DL and 7DS were 43, 78 and 48, respectively (Additional file 9: Table S7). Notably, one up-regulated *SBE2* gene (*TRIAE_CS42_2DL_TGACv1_158200_AA0512220*) in resynthesized relative to natural allohexaploid wheat was located within the QTL region on chromosome 2DL. In addition, one up-regulated *GATL* (*Galacturonosyltransferase-Like*) gene (*TRIAE_CS42_7DS_TGACv1_ 622210_AA2035360*) in resynthesized relative to natural allohexaploid wheat within the QTL region on chromosome 7DL was involved in cell wall biosynthesis [66]. Collectively, these genes are possible candidates for the detected QTL controlling grain size and shape, and detailed studies would be necessary to evaluate the relationship of QTL to differentially expressed genes identified in the present study.

Up to date, some wheat genes controlling grain size and weight were successfully isolated by the homology-based approach, including *TaCwi-A1*, *TaGW2*, *TaTGW6*, *TaGS1a*, *TaGASR7-A1*, *TaCYP78A3*, *TaCWI*, *TaCKX6-D1* and *TaSAP1-A1* [9–15, 67–69]. However, none of these genes were differentially expressed between 6-DAP grains of the natural allohexaploid TAA10 and resynthesized allohexaploid wheat XX329 (Additional file 9: Table S7). In addition, although a large number of up-regulated genes (362) in resynthesized relative to the natural allohexaploid wheat were overrepresented in the genomic region on chromosome 1AL, to the best of our knowledge, no known QTL controlling grain size and weight matched to the genomic region on chromosome 1AL that enriched differentially expressed genes, which deserve further investigation.

## Conclusions

Significant differences in grain size and weight at the early stages of development were observed between the natural and resynthesized allohexaploid wheats (TAA10 and XX329), which could be mainly attributed to the higher growth rates of the pericarp and endosperm cells in XX329 compared to TAA10. Comparative transcriptome analysis indicated that the differentially expressed genes were significantly enriched in the functional categories of cell wall, carbohydrate and hormone metabolism, which may play important roles in the observed difference between TAA10 and XX329 in terms of grain size and weight. Notably, consistent with the up-regulation of sucrose synthase genes in resynthesized relative to natural allohexaploid wheat, the resynthesized allohexaploid wheat showed higher contents of glucose and fructose in grains at 6 DAP than those of the natural allohexaploid wheat, indicating that alteration of sucrose metabolism was essential for the formation of large grains in the resynthesized allohexaploid wheat.

## Additional files

Additional file 1: Figure S1. Correlation coefficients among the three biological replicates of TAA10 and XX329 by Pearson correlation analysis. (XLSX 2028 kb)

Additional file 2: Table S1. Detailed information of the 8891 differentially expressed genes between the natural and resynthesized allohexaploid wheats (TAA10 and XX329). (XLSX 473 kb)

Additional file 3: Table S6. Detailed information of genes used for qRT-PCR analysis. (XLSX 69 kb)

Additional file 4: Table S2. The MapMan functional categories of the differentially expressed genes between the natural and resynthesized allohexaploid wheats (TAA10 and XX329). (XLSX 27 kb)

Additional file 5: Table S3. The MapMan enrichment analysis of the differentially expressed genes between the natural and resynthesized allohexaploid wheats (TAA10 and XX329). (XLSX 22 kb)

Additional file 6: Figure S2. Verification of 5 differentially expressed genes involved in carbohydrate metabolism by qRT-PCR. (XLSX 12 kb)

Additional file 7: Table S4. The differentially expressed genes in the enriched MapMan categories of cell wall metabolism. (XLSX 33 kb)

Additional file 8: Table S5. The differentially expressed genes in the enriched MapMan categories of hormone metabolism. (DOCX 156 kb)

Additional file 9: Table S7. Detailed information of genes associated with known QTL and candidate genes controlling wheat grain size and weight. (DOCX 143 kb)

## Abbreviations

AGPase: ADP-glucose pyrophosphorylase
DAP: Days after pollination
DBE: Starch debranching enzyme
FDR: False discovery rate
FPKM: Fragments per kilobase per Million reads
ILR: IAA-leucine-resistant
PCR: Polymerase chain reaction
QRT-PCR: Quantitative reverse transcription PCR
SBE: Starch branching enzyme
SS: Starch synthase
SUS: Sucrose synthase
TGW: Thousand grain weight
XTH: Xyloglucan transglucosylase/hydrolase
