# Supplementary figures and images for: Transcriptome analysis reveals potential mechanisms for different grain size between natural and resynthesized allohexaploid wheats with near-identical AABB genomes
Source: BMC Plant Biol. 2018 Feb 5;18:28. doi: 10.1186/s12870-018-1248-y (PMC5799976; doi:10.1186/s12870-018-1248-y)

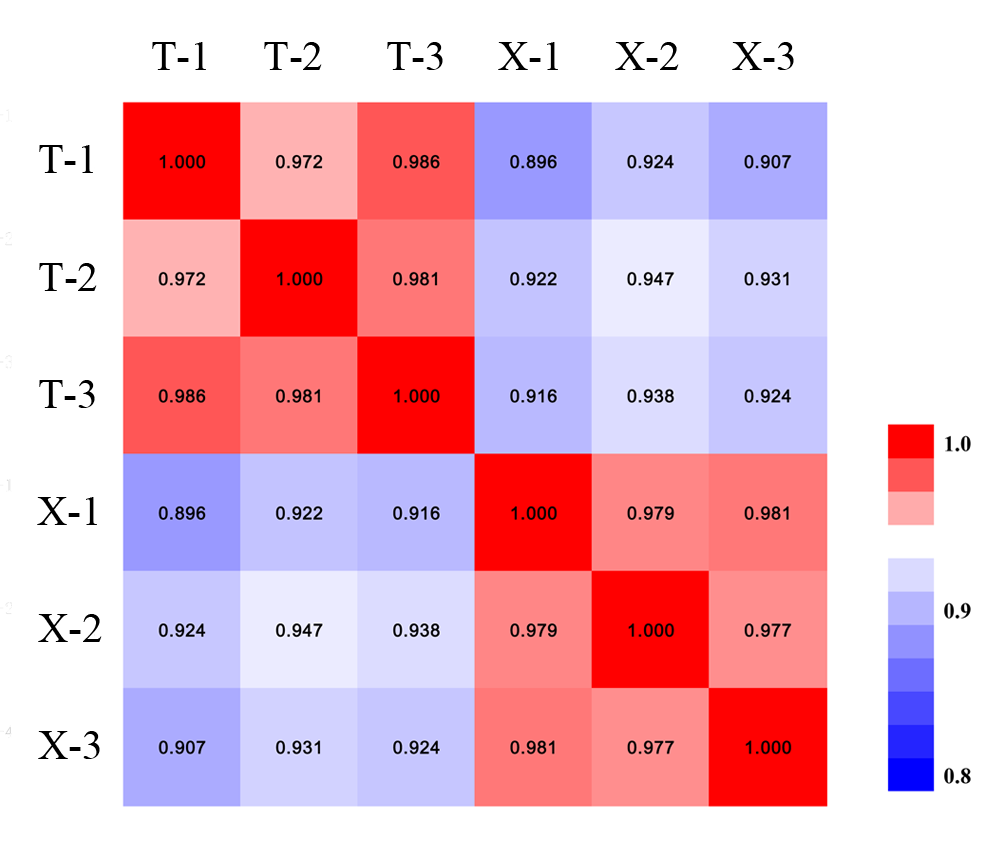

Supplement: Supplementary file 8 — The differentially expressed genes in the enriched MapMan categories of hormone metabolism. (DOCX 156 kb) [file 12870_2018_1248_MOESM8_ESM.docx]

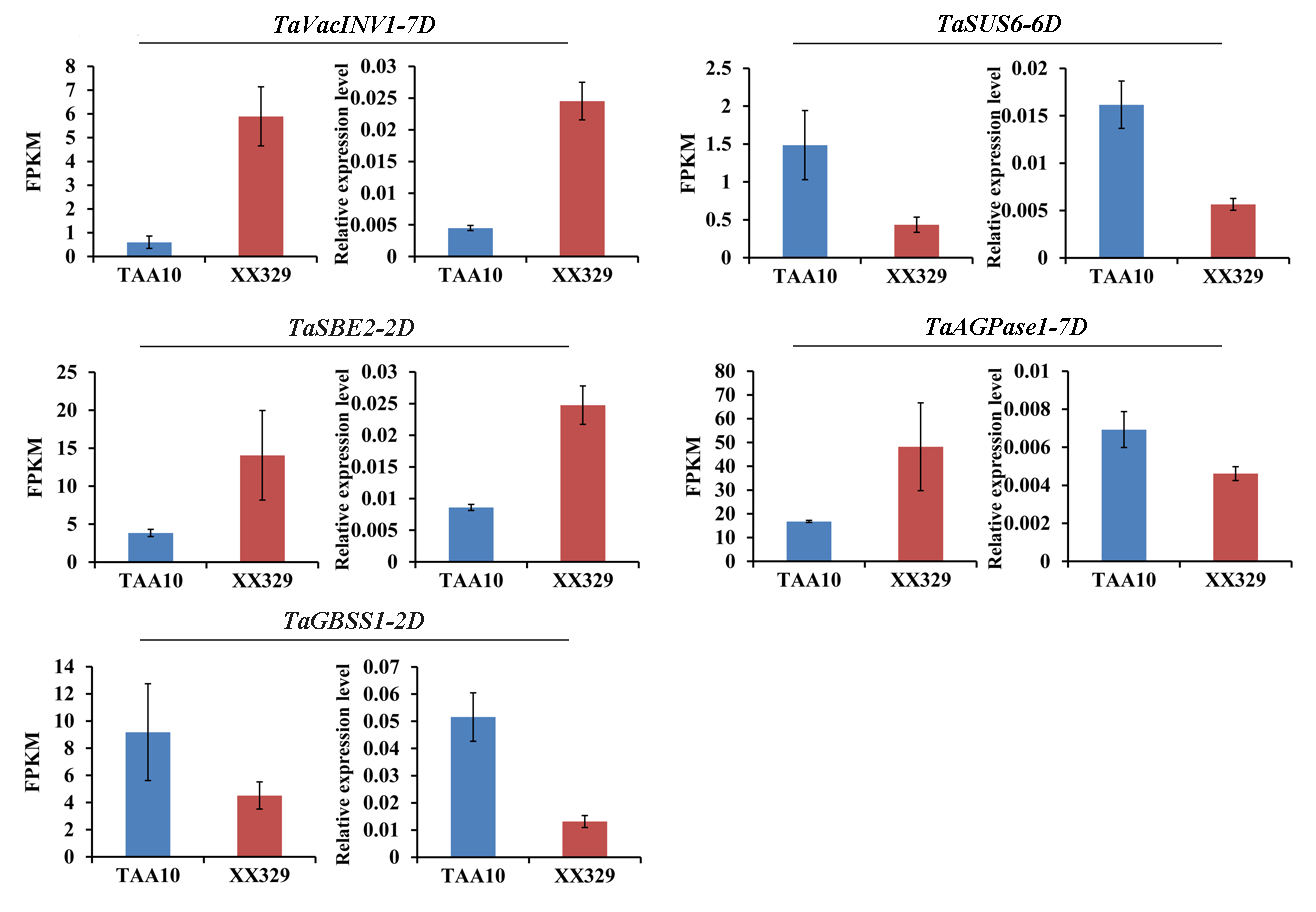

Supplement: Supplementary file 9 — Detailed information of genes associated with known QTL and candidate genes controlling wheat grain size and weight. (DOCX 143 kb) [file 12870_2018_1248_MOESM9_ESM.docx]
